# Proposed Physiological and Neurobiological Mechanisms of Music’s Effect, with a Focus on the Perioperative Period: Literature Evidence from Human, Canine and Feline Medicine

**DOI:** 10.3390/vetsci12080770

**Published:** 2025-08-17

**Authors:** Stefanos G. Georgiou, Apostolos D. Galatos

**Affiliations:** Clinic of Surgery, Faculty of Veterinary Science, School of Health Sciences, University of Thessaly, 43100 Karditsa, Greece; stegeorgiou@uth.gr

**Keywords:** dog, cat, physiology, stress, anxiety, perioperative period, anaesthesia, analgesia, music

## Abstract

There is growing evidence regarding non-pharmacological therapies such as music as a supportive approach for the treatment of various clinical conditions in humans. Physiological and neurobiological research suggests that music exposure is related to endorphin, endocannabinoid and dopamine release, favourable effects on autonomic nervous system functioning and is associated with decreased pain perception and reduced stress response. Further evidence in humans demonstrates a beneficial role of music application during the perioperative period by improving various outcome measures, such as the perioperative stress and anxiety levels, the sedation or general anaesthetic requirements, the pain levels, the analgesic requirements and other parameters related to patient prognosis, without reported side effects. Accordingly, such interventions have been considered as a method of environmental enrichment for animal welfare enhancement, by masking potentially disturbing background noises and by ameliorating anxiety or aggressive behaviours in different stressful settings in dogs and cats. Furthermore, research has been lately extended to the potential music’s effect in these species during the perioperative period, considered a stressful setting, as well. This review presents the existing evidence of music application focusing on the perioperative period of dogs and cats, as part of a multimodal approach, to improve their surgical outcome and welfare.

## Simple Summary

Music therapy has emerged as a promising non-pharmacological intervention in the perioperative setting, demonstrating efficacy in a variety of patient outcomes in humans, and many authors propose that perioperative music interventions should be available to all patients undergoing surgical operations. Considering that literature regarding the perioperative music application in dogs and cats is limited and rather inconsistent, this study aims to draw some preliminary conclusions in that context, by evaluating current evidence of music implementation as a means of welfare enhancement and by incorporating evidence from human medicine, when required. A review of the physiological underpinnings of music application and the proposed neurobiological mechanisms of its effect, the music’s impact on parameters related to animal welfare, and the effect of music during the perioperative period in dogs and cats, according to the existing literature, is being conducted. The current review underscores the potential role of music incorporation into the perioperative period in dogs and cats as a safe, low-cost, low-risk, non-invasive, non-pharmacological adjunct to conventional procedures and pharmacotherapies, as part of a multimodal approach, to improve their surgical outcome and welfare.

## 1. Introduction

Music has been a fundamental segment of human life almost since the first steps of our species and the interplay between music and medicine has been investigated lately in several studies. The findings on the potential health benefits of music in people seem to be promising, albeit not consistent [1]. There is growing literature evidence of its therapeutic applications in humans during recent years. This evidence highlights the potential of music therapy as a supportive approach in the treatment of various clinical conditions, including hypertension [2], epilepsy [3,4,5], anxiety, depression, dementia, Parkinson’s disease [1,4], acute and chronic pain [6,7,8,9] and cancer [1,10]. Further evidence exists of the potential beneficial role of music application during the perioperative period in humans by improving postoperative outcomes in various surgical operations [11,12,13,14,15,16,17,18].

Anxiety and insufficient management of perioperative pain have been connected to numerous negative outcomes in humans, like undesirable haemodynamic incidents because of sympathetic, parasympathetic and endocrine stimulation, increased anaesthetic and analgesic requirements, delayed recovery, delayed wound healing, extended length of hospitalization, compromised immune system function and increased morbidity [19,20,21]. The same has been proposed for cats, as stress responses triggered by protective emotions stimulate the release of catecholamines and the ensuing physiological changes can elevate the anaesthesia-related risk [22]. Furthermore, according to recent guidelines in the context of producing a cat-friendly environment in a clinical setting, stress events during the perioperative period can increase complications, thus influencing wound healing and exacerbating postoperative pain, which apart from humans seems to apply in cats’ and dogs’ perioperative period, as well [23].

Opioids still hold a prominent position regarding intraoperative analgesia and postoperative pain management for moderate to severe pain in humans [7,17,19,24], and, similarly, are commonly used for acute pain management in dogs and cats [25]; however, multimodal analgesia has become a fundamental strategy for perioperative pain management [7,19,24]. The term multimodal analgesia was first introduced about 20 years ago and refers to the synergistic effects of pharmacological agents with different modes or sites of action throughout the pain pathway, such as regional anaesthesia, opioid and non-opioid analgesics, nonsteroidal anti-inflammatory drugs and variable adjuvant agents [26]. Many of the existing clinical practice guidelines recommend the use of multimodal analgesia to improve perioperative pain management and minimize opioid-related adverse effects, in combination with non-pharmacological and non-opioid modalities [24]. Recent guidelines for the recognition, assessment and treatment of pain in animals recommend the use of multimodal approaches in all animals’ surgical cases [25].

Furthermore, there is growing interest in non-pharmacological approaches, in terms of integrative medicine, as an adjunct to conventional procedures and pharmacotherapies and their potential effect on the perioperative period, as part of a multimodal approach. Music-based interventions, as a non-pharmacological adjunct, seem to be an effective approach for the perioperative control of pain and anxiety and sedative requirement reduction in surgical patients [7,11,12,14,17,18,27,28]. More specifically, the association between music therapy and pain has been studied in a wide variety of surgeries in humans, including breast, cardiothoracic, orthopaedic and abdominal [12,13,14,15,16,17,18,28].

There is also recent research interest in how music interventions may enhance animal welfare and overall well-being. In that context, music application has been investigated as an environmental enrichment method to ameliorate signs of anxiety and stress in kenneled dogs [29,30,31,32,33,34,35,36], in dogs [37,38] and cats [39] during a veterinary clinic visit, and in hospitalized cats [40]. Furthermore, research has been lately extended to the potential music’s effect during the perioperative period in dogs and cats [41,42,43,44,45,46], which is thought to be a stressful setting such as the hospital or the kenneled environment.

Considering that music treatment is a safe, low-cost, low-risk intervention that is easy to implement additionally to conventional pharmacological approaches, and in the light of encouraging preliminary literature reports, a role may arise for music as a noninvasive complementary tool during the perioperative period to potentially improve surgical outcome and welfare in dogs and cats.

However, the components and the characteristics of music should be standardized for a successful perioperative intervention in dogs and cats. To be more specific, music choice (type of music, music genre), music instrumentation, inclusion of vocal music or human voice, duration of exposure throughout the intervention, the phase of the perioperative period, pitch (particular frequency bands), tempo (rhythm), sound volume level [decibel (dB)], delivery methods (loudspeakers, headphones), the ability of a particular animal species to perceive the particular stimulus and the defined outcome measures remain inconsistent and are among the challenges [30,31,38,46].

It is evident in veterinary literature that the impact of music on different patient populations can vary, owing to factors such as the music exposure methods, and the type and severity of the medical condition. In that context, assumptions are often derived from studies involving healthy animals which may not reflect the conditions of hospitalized patients or those in clinical settings, while sample sizes are typically small, with limited randomization and varying treatment methods [38,47]. Although some generalizations can be inferred from human literature, where positive effects of music-based interventions have been reported, the high degree of heterogeneity even in human study designs has led to inconsistent conclusions and the effectiveness of such interventions has not been fully elucidated [1,28]. Indeed, that seems to apply to studies in dogs and cats as well. Some studies in various clinical settings with different primary outcomes have shown music’s beneficial effects in dogs [29,30,31,33,34,35,44,45,46,48,49] and cats [39,40,41,42,50], while others have shown that it has limited or no effect [32,37,38,43].

The basic knowledge of how music affects physiology and the suggested neurobiological mechanisms involved in musical processing in the brain are presented in the current review. A further objective is to provide the existing evidence of music implementation, either as an environmental enrichment method or during the perioperative period, and its proposed effects on anxiety, anaesthesia and analgesia as a non-pharmacological adjunct both in humans and companion animals.

## 2. Methods

This manuscript presents a narrative review of the current literature with a focus on the use of music therapy during the perioperative period in dogs and cats. Relevant literature was identified through targeted searches of databases such as PubMed, Scopus and Google Scholar using combinations of keywords such as “music therapy”, “physiology”, “neurobiology”, “stress”, “anxiety”, perioperative”, “anaesthesia”, “dogs” and “cats”. Reference lists of relevant studies and reviews were also assessed to identify additional sources. Studies were included based on their relevance to the topic and their contribution to understanding the proposed effects of music on physiological, neurobiological or behavioural parameters which could be applied during the perioperative period in companion animals. No strict inclusion or exclusion criteria were applied, allowing for the inclusion of diverse perspectives and study types. This flexible and integrative approach was considered appropriate to capture the conceptual scope of this emerging research area.

## 3. Physiology of Music Application and Proposed Neurobiological Mechanisms of Its Effect

**Figure 1:**
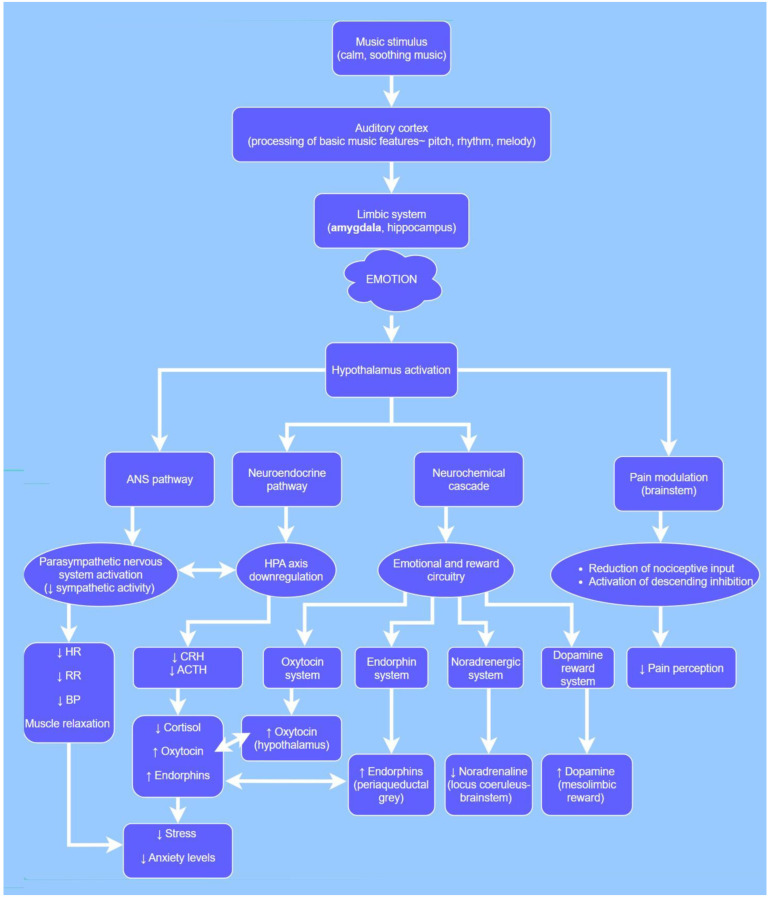
Proposed neurobiological pathways mediating the effects of music in dogs and cats. Most pathways are extrapolated from human literature due to limited veterinary data. Specific species differences (e.g., auditory range, music preferences) may modify these responses and warrant further investigation. ↓ = decreased; ↑ = increased. ANS: autonomic nervous system, HPA axis: hypothalamic-pituitary–adrenal axis, HR: heart rate, RR: respiratory rate, BP: blood pressure, CRH: corticotropin-releasing hormone, ACTH: adrenocorticotropic hormone.

Several evidence-based physiological and neurobiological mechanisms have been attributed to music exposure, in a variety of settings, with a focus on its potential effect on the perioperative period. The proposed mechanisms originated from studies in both healthy and diseased human individuals and non-human subjects in a variety of clinical conditions (Figure 1).

The neural mechanisms involved in music processing and perception in the brain have been evaluated in a variety of studies using functional neuroimaging methods like electroencephalography (EEG), magnetoencephalography (MEG), positron emission tomography (PET) and functional magnetic resonance imaging (fMRI) [51,52,53,54,55,56,57,58,59,60].

### 3.1. Neurobiological Pathways Mediating Music’s Effects in Human Medicine

The neural networks that are associated with music processing in humans are widespread, beginning in the peripheral auditory apparatus (cochlea) and projecting through various brainstem nuclei to the auditory midbrain (inferior colliculus), and from there to the auditory cortex (AC) in the temporal lobe [60]. Various regions of the cerebral cortex, primarily in the right hemisphere, are responsible for encoding distinct music features such as pitch, rhythm or intensity with evidence supporting the AC’s role in processing these fundamental auditory features [52,55,60]. Research from fMRI and PET studies suggests that the AC and other temporal lobe regions, particularly in the right hemisphere, are activated during passive listening and respond to subtle changes in pitch and timbre [51,61,62,63]. In contrast, the perception of temporal acoustic signals like rhythm appears to engage the AC in a more left-lateralized or bilateral manner, likely involving both hemispheres [51,55].

Music exposure can induce mental and physical effects on the human body, and although the exact underlying mechanisms of music’s effect are not clearly understood, a few potential theoretical frameworks have been proposed [1]. Musical stimuli have been shown to activate specific pathways in several brain areas associated with emotional behaviours, such as the insular and cingulate cortex, hypothalamus, hippocampus, amygdala and prefrontal cortex, meaning that the emotional processing of musical stimuli is not confined to subcortical regions, but is also cortically mediated [52]. Notably, brain activity was mainly present in the frontal lobes while humans listened to pleasant music, and in the temporal lobes when they were exposed to unpleasant music [64]. Furthermore, an fMRI study evaluating the neural correlates of emotion processing in response to music found that unpleasant music activated the amygdala, hippocampus, parahippocampal gyrus and temporal lobes, structures playing an important role in the processing of stimuli with various degrees of negative emotional valence. Conversely, pleasant music has been found to activate the inferior frontal gyrus, inferior Brodmann’s area of the neocortex, anterior superior insula, ventral striatum, Heschl’s gyrus, as well as the Rolandic operculum. This suggests that these structures respond to both pleasant and unpleasant auditory stimuli with emotional significance, and that listening to music can both enhance and reduce neuronal activity in these regions [53].

The proposed alterations in neural pathways that music triggers in the cerebral cortex, hypothalamus, limbic system and insula may promote relaxation and result in physiological changes [27]. Furthermore, neurochemical research suggests that various neurotransmitters, neuropeptides and other biochemical mediators, such as endorphins, endocannabinoids and dopamine, may contribute to the brain’s perceptual and emotional processing of music [51,52].

Exposure to music produces beneficial effects on autonomic nervous system (ANS) functioning, inducing adaptive changes in heart rate variability (HRV), heart and respiratory rates, electrodermal activity, skin temperature and locomotion [12,51,52,55,65,66,67]. The ANS is responsible for regulating internal physiological functions, such as blood pressure (BP), heart rate (HR), digestion and respiration. It consists of the sympathetic and parasympathetic systems, which work together to maintain homeostasis often exerting opposing effects. The sympathetic system typically increases HR and BP, while the parasympathetic system works to decrease them [60]. Sympathetic activation appears to drive various physiological signs related to anxiety, such as tachycardia, sweating or flushing. In healthy individuals, anxiety has been linked to marked increases in both BP and plasma noradrenaline levels [68]. Music can decrease sympathetic activity, and thus anxiety and stress [12,14,65,69,70], and seems to enhance the parasympathetic tone by reducing plasma cytokine and catecholamine levels like adrenaline and noradrenaline [71]. HRV is a useful indicator of the balance between sympathetic and parasympathetic nervous system activity. Research on music-based interventions has shown that they can increase HRV and decrease HR and BP, reflecting increased vagal tone and enhanced activation of the parasympathetic nervous system [12,60,65,66,67,72,73].

The effects of music on autonomic regulation are often associated with decreased pain perception and a reduced stress response and are commonly accompanied by lower levels of cortisol, a hormone related to stress [15,73,74,75], and an increase in oxytocin levels [65,73,76]. Oxytocin has notable anti-stress and anti-anxiety properties and may enhance vagal activity by suppressing sympathetic nervous system (SNS) responses and hypothalamic–pituitary–adrenal (HPA) axis activation during stress, suggesting that higher oxytocin levels promote relaxation through vagal nerve stimulation [73,76]. Brain regions involved in stress response, namely, the prefrontal cortex, amygdala and hippocampus, are also involved in pain processing [77]. These, in turn, are closely connected to the hypothalamus and thereby influence the HPA axis and the SNS by transmitting signals to the adrenal glands [78].

There is evidence that some of the antinociceptive effects of music are mediated in the periphery and at spinal and brainstem levels, involving both ascending and descending systems, and potentially involving a variable combination of β-endorphin, oxytocin, dopamine, noradrenaline and/or serotonin interactions [9,60]. It seems that connectivity between the AC, the amygdala and plenty of other regions involved in emotional processing allows for modulation of stimulus perception and is further involved in the neurobiology of acute and chronic pain [8,60,78,79,80]. In the same context, a link has been identified between the desire to listen to a pleasurable song and an increase in circulating endogenous opioids, along with enhanced expression of the mu-opioid receptor [81]. Moreover, music that is perceived as pleasant can activate a dopamine-driven reward cascade in the central nervous system [12,51,54,66], whereas unpleasant music has been associated with increased pain perception compared to pleasant [79,82].

Neuroscientific evidence also supports the idea that music can influence pain pathways. Neuroimaging studies have been used to investigate and shed light on the mechanisms behind music’s analgesic effects, leading to the development of several proposed theoretical frameworks. Furthermore, the potential role of neurotransmitters in music-induced analgesia is mainly based on studies that associate the enjoyment of music listening with the release of endogenous opioids and dopamine, using PET imaging and pharmacological approaches involving receptor agonists and antagonists [51,54,81,83,84].

Lu et al. [57] used EEG to assess brain activity in participants who listened to self-selected music just before application of a painful stimulus and observed a decrease in alpha oscillations. This reduction corresponded to the activation of brain regions involved in music processing, primarily the prefrontal cortex and anterior cingulate cortex (ACC). These findings indicate that music may modulate pain, particularly by reducing the pain unpleasantness induced through its positive emotional effects, further hypothesizing that music activates brain areas involved in the descending pain pathways [57]. Additionally, two studies identified several key regions associated with music-induced analgesia using fMRI. In the first study, the researchers assessed brain responses to painful thermal stimuli while participants listened to their favourite music. They observed activation not only in brain areas associated with the pleasure of music, such as the amygdala, hippocampus and nucleus accumbens, but also in areas involved in the descending pain modulatory pathway, including the periaqueductal gray (PAG), rostral ventromedial medulla and dorsal gray matter of the spinal cord [56]. Similarly, Antioch et al. [58] suggested that listening to preferred music, as opposed to resting in silence, modulated brain responses to electrical stimuli in the ACC, an area associated with emotion. Considering that music activates neural pathways associated with reward and pleasure [51,53,85] and that pleasant music can trigger dopamine release [54], these findings strongly suggest that music can influence pain processing through emotional modulation. Furthermore, given the central role of the PAG in the descending pain modulatory system, along with its high concentration of endogenous opioids and opioid receptors, these findings support the hypothesis that music activates descending pain modulation by promoting endogenous opioid release and suppressing activity in the ascending pain pathways [56,58].

### 3.2. Proposed Theoretical Frameworks Justifying the Use of Music in Human Medicine

A few theoretical frameworks have been proposed that have been used as an underlying mechanism to explain how music induces an analgesic effect. Williams & Hine [70] found that across 39 papers, distraction (in terms of gate control theory) was mostly used as a potential mechanism of music’s analgesic effect, followed by relaxation, oscillatory entrainment of neuronal activity, emotional shift and endogenous analgesics stimulated by music [78]. Distraction was the most popular theory used to describe the effect of music in the studies of the respective review [70]. Regarding that theory, music acts as a pleasant diversion, by distracting the patients or blocking the noise from the stressful environment of the hospital. The mechanism seems to align with the gate control theory [86], in which music serves as a competing sensory input that effectively closes the “gate”, inhibiting the transmission of pain signals to the brain [66,70]. Relaxation was another theory proposed in 35% of studies. The positive effect of music was attributed to relaxation induced either by providing mental focus, by promoting the relaxation of tense muscles, or by stimulating alpha waves, which lead to the release of endorphins [70]. Entrainment, featured as a theory in 15.4% of studies, is described as the synchronization of oscillating rhythms. It was proposed that music can cause a shift in neuronal activity in the lateral temporal lobe and cortical areas devoted to movement, which subconsciously allows the patients’ bodily rhythms (e.g., RR) to synchronize with the rhythm of the music. Entrainment may lead to a decrease in sympathetic nervous system activity by decreasing adrenergic activity, neuromuscular arousal, cardiovascular and respiratory rates, tension and metabolic rate, among others [70]. The theory of music-induced analgesia via the release of endogenous analgesics was only present in 7.7% of the studies. According to these studies, music can mediate mu opiate receptor expression, morphine-6 glucuronide and interleukin-6 levels, and increase endorphin levels, which agonize the opiate receptors, reducing pain sensation and the need for analgesics [66,70]. The impact of music in reducing pain can be attributed to its ability to increase the release of dopamine and endogenous opioids, which exert antinociceptive properties [56,58]. The final theory proposed, stated in 23.1% of the studies, was attributed to music’s ability to alter emotional states positively, by reducing stress and anxiety [70]. In addition, self-selected favourite music strongly reduced pain intensity and unpleasantness compared to silence [87]. Additionally, a more recent review proposed a literature-based framework constituted of three main components: cognition, emotion and neurobiology, meaning cognitive distraction (music-induced analgesia by diverting the patient’s attention to a competing stimulus), music-induced emotion and neurotransmitters (endogenous opioids, endogenous dopamine) [88].

However, some challenges exist, and these proposed theoretical frameworks are still under consideration, while the exact psychological mechanisms of music-induced analgesia remain unknown [89]. A recent randomized study involving healthy participants found no significant increase in pain tolerance in the group passively exposed to music compared to the silence group; however, the authors noted that engaging in a task, as a form of distraction, could potentially enhance pain tolerance [89]. In the same context, there are studies that argue that the pain modulatory effects of music are not merely due to attentional distraction [57,79,82,87,90]. Furthermore, music still reduces outcome measures of pain even when the patients are under general anaesthesia (or just during the immediate postoperative period), with that being impossible to be attributed to music’s distractive effect [12,14,91,92]. Additionally, with regard to the proposed neurobiological underpinnings of music-induced analgesia (endogenous opioids and dopamine), Lunde et al. [93] observed that even when oral opioid (naltrexone) or dopamine (haloperidol) antagonists were administered in healthy subjects during music exposure, an analgesic effect of music was still apparent and neither of the antagonists attenuated music’s effect. According to the authors, the most likely explanation for that result was the participants’ expectation for pain relief, potentially exhibiting a placebo effect, rather than an opioid- or dopamine-dependent effect [93,94]; however, it should be noted that the music was not self-chosen, which seems to be of importance. The lack of effect of the dopamine antagonist in the previous study may be attributed to the type of music (not self-selected), if we consider the established role of dopamine in the rewarding aspects of pleasurable music listening [51,54,84]. Concerning the lack of effect of the opioid antagonist in the same study, it could be attributed to the fact that the endogenous mu opioid signaling is not necessary for subjective enjoyment of music; this has been observed again as opioid blockers administered while listening to music did not change the subjective ratings of music-induced pleasure [85,95], while others reported a reduction in subjective behavioural measures of pleasure after the administration of naloxone or naltrexone [81,83].

Regarding the neurobiological underpinnings of music-induced analgesia, although the endogenous opioid and dopamine-dependent mechanisms have been proposed to play a role, a direct link has not been established as our current knowledge on underlying neural activity derives mostly from brain imaging studies. Although fMRI BOLD responses can represent underlying neural activity, they do not directly reflect neurotransmitter activity [84]. Therefore, further evidence is needed to demonstrate that music activates the descending pain modulatory system through the endogenous opioid and dopamine release [93]. Thus, more pharmacological antagonist studies are needed to further investigate that hypothesis [88], and also the potential involvement of placebo mechanisms in music’s effect should be assessed [93]. Although the potential distractive effect and placebo effect of music should not be ignored, perioperative music interventions during general anaesthesia did show a statistically significant pain-reducing effect [12,14], not corresponding with a mere distractive or placebo effect.

### 3.3. Evidence Deriving from Animal Studies

There have been attempts to gather and describe the potential underlying mechanisms that music’s effect is attributed to, in terms of welfare, according to animal studies. Firstly, the concept of masking aversive or stressful sounds (acoustic masking hypothesis) seems to conform with the theoretical framework of distraction that has been proposed for humans. This hypothesis insinuates that animals may perceive music as another form of noise, that is perhaps more tolerable. However, as in humans, there is evidence that music may have effects beyond just masking noise, as different types of music seem to affect behaviour and physiology in different ways [96,97]. The second proposed mechanism is the sensory stimulation hypothesis, and the third proposed mechanism is arousal modulation [98].

Although there are no neuroimaging or neurochemical studies in dogs and cats to evaluate the impact of music on different brain regions or biochemical mediators, or to investigate the underlying mechanisms of its effect, there are a few studies in rodents trying to explain these mechanisms of music’s potentially beneficial effect. These changes in physiology, cognition and brain chemistry, although not widely studied in animal models, could provide some evidence that music may affect animals similarly to humans. Exposure to Mozart’s music resulted in reduced systolic BP in spontaneously hypertensive rats, and it was observed that music significantly increased serum calcium levels and dopamine levels in the lateral neostriatum region of the brain. The BP reduction was attributed to a calcium-dependent dopamine synthesis in the brain, and in turn, the enhanced dopaminergic activity inhibited sympathetic nerve activity (via D_2_ receptors), resulting in decreased BP. So, the authors proposed that music may regulate or affect various brain functions in a dopaminergic neurotransmission manner [99]. Another study by the same team showed that the most significant BP-reducing effect in spontaneously hypertensive rats was observed at 4–16 kilohertz (kHz) compared with lower frequencies, and this was attributed to a greater dopamine synthesis stimulation [100]. In the same context, Lemmer et al. [101] demonstrated that Mozart’s music significantly decreased HR, although no effect was observed on BP in spontaneously hypertensive rats.

Physiological results from studies in dogs have reported that classical music significantly lowered HR and elicited changes in HRV indicative of parasympathetic nervous system dominance [31,33]. As proposed in human medicine, a useful measure of sympathetic–parasympathetic balance is HRV, RR and changes in cardiovascular signs [12,60,65,66,67,72,73]. In the study of Bowman et al. [31], measurement of HRV, HR and salivary cortisol concentrations were employed to assess changes in the ANS activity and HPA axis and the authors concluded that these results reflect a true physiological/psychological response to the music stimulation, thus, indicating an up-regulation of the parasympathetic component of the ANS in dogs within a stressful kennel environment. A similar observation was reported in another canine study where dogs in a similar stressful environment exhibited increased levels of HRV parameters during exposure to different genres of music, reflecting an increase in parasympathetic nervous system dominance [33].

Finally, a number of studies in dogs investigated parameters that could be associated with an, either beneficial or not, effect on ANS after music exposure; however, the study did not explain the assumed underlying physiological mechanism for that response. The body temperature and HR were not different in dogs exposed to classical music during a veterinary visit [37]. The same was observed for HR and RR in another study; however, the core body temperature was significantly lower in the classical music group [38]. Finally, Koster et al. [34] reported that classical music may exhibit an excitatory rather than a calming effect, according to HRV measurement.

Although the musical features of the selected musical pieces on the conducted canine studies, thus far, were not attributed to a specific underlying framework, these miscellaneous data may imply that music can exert physiological effects and affect animals similarly to humans, with this impact potentially being extended to the perioperative period.

## 4. Music’s Effect on Stress and Anxiety

### 4.1. Literature Evidence on Humans

Music interventions have been reported to exhibit a stress- and anxiety-reducing effect on both healthy and diseased humans. Sixteen healthy individuals exhibited a significant reduction in anxiety levels, as defined by a dramatic decrease in both systolic and diastolic BP, when exposed to their preferred music for 20 min [65]. In another study, music listening induced relaxation in 26 healthy participants when they were exposed to 20 min slow-tempo piano musical pieces composed by Chopin. Considering that oxytocin has anti-stress and anti-anxiety effects, the participants’ salivary oxytocin levels after music exposure were increased, indicating parasympathetic nerve activity through a vagal-mediated relaxation effect [73].

Furthermore, music therapy as an adjunctive tool has been reported to improve human patient outcomes in a variety of clinical conditions. Music listening contributed to reduced stress levels in patients waiting for radiotherapy [102], while music interventions have been associated with noteworthy anxiety- and stress-reducing effects in patients with cancer [10], in heart disease patients [2,11,55,71,72] and in patients with chronic pain [103,104]. A systematic review including 17 studies and 1381 participants demonstrated a large anxiety-reducing effect of music interventions in adult cancer patients [10]. Regarding heart disease patients, music therapy can reduce anxiety [11,72] which was a rather consistent finding in the studies evaluated, and in addition, the effect was associated with lower HR and BP measurements [72]. In a study of 87 elderly patients with cerebrovascular disease and dementia, music therapy enhanced parasympathetic activities and decreased congestive heart failure (CHF) events by reducing plasma cytokine and catecholamine levels. Specifically, patients who received 45 min music therapy sessions at least weekly for 10 months had decreased anxiety, tachycardia and tachypnoea and significantly lower incidents of CHF events, by reducing adrenaline and noradrenaline levels, compared to patients not being monitored by a music therapist [71]. Furthermore, daily music listening has been suggested to promote cognitive recovery after stroke by alleviating the anxiety and psychological stress experienced by those patients [55]. It has been proposed that along with high BP, anxiety, depression and sleep disorders are very common in patients with hypertension, affecting the degree of hypertension and reducing their quality of life. A recent meta-analysis in hypertensive patients (20 RCTs including 2306 patients) concluded that music therapy, as an adjunct to routine anti-hypertension treatment, is beneficial in reducing the systolic and diastolic BP and HR, and is helpful in reducing anxiety, depression levels and improving the sleep quality of those patients [2].

Anxiety has been reported to be a common comorbidity in chronic pain. A meta-analysis of 14 RCTs showed that music reduces chronic pain, as well as anxiety and depression, regardless of the etiology of the pain. The studies included patients diagnosed with cancer pain, fibromyalgia, osteoarthritis, multiple sclerosis, inflammatory bowel disease or palliative care patients [103]. In the same context, there has been a report of a woman who has lived 20 years with chronic pain and music listening contributed to a significant improvement in quality of life. Apart from pain relief and minimization of withdrawal effects after discontinuing her opioid-based treatment, she reported a significant reduction in depression and anxiety symptoms and an improvement in sleep quality [104].

### 4.2. Literature Evidence on Dogs

In accordance with human studies regarding the anxiety- and stress-reducing effect of music, such interventions have been considered as a method of environmental (auditory) enrichment for improving animal welfare, by masking potentially disturbing background noises, and by decreasing anxiety, stress or aggressive behaviours [96]. Shelters, laboratories, university facilities, veterinary clinics and boarding kennels are environments that can be loud and unpredictable, and thus stressful and challenging for dogs and cats. This can lead to high levels of stress and arousal, contributing to behavioural changes and negatively affecting their welfare [36,105]. Among others, increased activity, vocalization (barking), panting, reduced time lying down/resting and body shaking have been reported as behaviours suggestive of anxiety and nervousness/stress in dogs [29,30,31,33,35,98,105,106], while cats were more likely to exhibit protective and hiding behaviours, unwillingness to handling and reduced interaction in the stressful veterinary clinic environment [23,39,40,107]. On the other hand, physiological parameters to evaluate stress in dogs include immune functions, HR, RR, arterial BP, SNS monitoring (HRV) and hormonal indicators (cortisol) [29,31,33,106,108]. In cats, significant physiological changes, such as elevations in BP, HR, RR, rectal body temperature and blood glucose were observed in a stressful environment, compared to the home environment [107,109]. Music enrichment in environments that are considered to be stressful, like shelters or veterinary hospitals, has been reported to promote behaviours and physiological responses associated with reduced stress levels both in dogs [29,30,31,32,33,35,48,49] and cats [39,40,50].

Sound levels in kenneled environments have been shown to exceed 100 dB [110,111]. Additionally, confinement in animal shelters, even for short periods, is a potential psychogenic stressor for most dogs and seems to produce a prolonged activation of the HPA axis [30,112]. Many dogs may display signs of acute stress, and in addition, plasma cortisol concentrations have been reported to be above the normal range, in some cases even three times greater, compared to those of household pets [112].

Results from studies in shelter dog populations have shown that auditory enrichment in the form of music may induce more relaxed and desirable responses, with these results potentially depending on the type of musical stimuli [29,30,31,33,35]. Kenneled dogs seem to exhibit calmer and more relaxed behaviours when exposed to classical music compared to heavy metal, rock, pop music, psychoacoustically designed dog music, human conversations or silence/no music [29,30,31,33]. When dogs listened to classical music, they spent more time resting and lying down quietly and less time standing and barking, compared to the other auditory stimuli or silence [29,30,31]. Furthermore, a more recent study implementing piano music (not characterized as classical music, though) found similar stress-attenuating effects, with kenneled dogs showing reduced arousal-related behaviours like spending more time lying down, and exhibiting fewer panting events, less time tail wagging and less vocalization, compared to a control condition [35]. Apart from classical music, there are results from two studies demonstrating that not only can classical music be effective in reducing stress and eliciting calm behaviours in kenneled dogs, but other music genres can also be potentially effective [33] or even audiobooks [32]. In the study of Bowman et al. [33], all different music genres (classical music, soft rock, Motown, pop, reggae) induced changes in behaviour indicating reduced stress (less time standing) compared to the silent condition, and dogs were more likely to bark following cessation of the auditory stimuli. Another study conducted in a rescue shelter used auditory stimulation in the form of audiobooks, classical music (Beethoven), pop music, psychoacoustically designed dog music and a control condition [32]. The authors observed that although classical music induced calmer behaviour than pop music and the silent condition, the audiobook treatment exhibited the most beneficial effects compared to all other treatments, even compared to Beethoven’s music.

Apart from music’s effect on behaviour, there are reports of its impact on physiological parameters which correlate with stress responses. Classical music induced changes in HRV in a way indicative of reduced stress compared to silence [31], but when classical music was compared to other music genres, HRV was found to be significantly higher, indicative of decreased stress, when dogs were exposed to soft rock and reggae, with a lesser effect observed for classical music [33]. Surprisingly though, in that study, cortisol was found to be higher during soft rock exposure compared to the other auditory stimuli [33].

However, there have been reports of dogs’ habituation to the effects of music if the same playlist is used repeatedly [31]. When dogs were exposed to classical music for 6.5 h per day for 7 days, the calming effects of music on HRV and behaviour that were observed on the 1st day were not maintained until day 7. This result suggests that the dogs may become refractory to these physiological/psychological effects of classical music when the same playlist is used repeatedly [31]. A later study by the same authors concluded that providing a variety of different genres mixed with classical music may help to overcome the potential habituation to music and maintain the degree of parasympathetic stimulation for a longer period. Auditory enrichment with a variety of different genres for 6 h per day for 5 days resulted in the preservation of the physiological and behavioural changes induced by music [33]. That was further supported by a more recent study in dogs in which piano music was provided to shelter dogs for 3 h per day for 5 consecutive days [35]. In that study, no evidence of habituation to the auditory stimuli was reported over the 5 days of exposure, as the treatment consisted of a 51-track selection provided with random order every day.

It has been proposed that a visit to a veterinary clinic (or hospitalization) can be as stressful as that of the kennel environment for dogs [38,108,113,114]. Stressors include exposure to unfamiliar surroundings, personnel and other dogs and may be exacerbated by separation from the owner. A study in 30 dogs reported that stress arising from transportation and environmental change during a veterinary clinic visit may contribute to sympathetic nervous system activation and alterations of vital signs [108]. More specifically, significant increases in rectal temperature, HR (mean increase of 11%), panting incidence, and systolic arterial BP (mean increase of 16%) were observed in the hospital environment, compared to the home environment [108]. It has also been suggested that moderate to severe anxiety and stress are strongly associated with physical examinations conducted away from the owner, with reversal of that state after transfer back to the owner [114], while one study reported that 106 out of 135 canine patients (78.5%) were fearful on the examination table [113]. Apart from the health examination procedure, it seems that being left alone in a kenneled area within a veterinary clinic, like during hospitalization, elicits different levels of stress for dogs [38].

Attempts have been made to evaluate the potential effect of music on stress and anxiety in a veterinary clinical setting; however, the results seem to be less clear compared to the shelter setting [34,37,38,48,49]. The only studies that reported some benefits of music on stress-related behaviours were those of Kinnaird et al. [48] and Guerineau et al. [49]. Dogs exposed to classical music (Mozart’s Sonata K.448) were significantly faster to lie down and quicker to settle than dogs in the audiobook and control conditions. The authors, however, concluded that classical music exhibited only a moderate calming effect compared to the existing literature for dogs in rescue shelters, while no benefits of an audiobook were observed on dogs separated from their owners [48]. In a more recent study, no significant behavioural responses were observed when dogs were exposed to classical music; however, subjects that were already familiar with classical music by listening at home exhibited more relaxed behaviours, that suggesting that familiarity may influence emotional responses [49]. On the other hand, no clear benefit of music listening in stress parameters was suggested by studies using bespoke music, i.e., designed to entrain physiological parameters of dogs, composed by a professional music producer [38], classical music [37] or psychoacoustically designed dog music (Through a Dog’s Ear, BioAcoustic Research, Inc., Jacksonville, FL, USA) [34,37]. In addition to that, Koster et al. [34] reported that according to HRV measurements, dog relaxation music had an excitatory rather than a calming effect. In any case, playing soothing background classical music and avoiding hard rock or heavy metal music has been recommended by guidelines for pet-friendly veterinary practices to maximize the environmental comfort of dogs [115].

### 4.3. Literature Evidence on Cats

Stress is another common predisposing factor that may complicate a veterinary visit for domestic cats. The fact that cats are more stressed in the veterinary clinic is supported by findings of significantly higher physiological parameters (BP, HR, RR) [109], higher blood glucose levels and more hiding behaviours in the clinic compared to the home environment [107]. Furthermore, temperature [109] and cortisol concentrations [107] were elevated in the clinic environment, albeit not significantly.

Reducing feline anxiety during veterinary visits could have benefits for domestic cats not only by reducing stress-related behaviours but also by facilitating in-clinic procedures. Recent guidelines for a cat-friendly manipulation of the veterinary clinic environment to minimize feline patients’ distress have proposed that playing calming classical music or cat-specific music may reduce acoustic stimuli and create a more relaxed atmosphere [23]. Indeed, there are reports that music can reduce stress in cats, both at home and in a veterinary clinic environment [39,40,50]. Snowdon et al. [50] concluded that cats, when exposed to music specifically designed for them, exhibited more positive behaviours than when human (classical) music was played in the home environment, suggesting that such auditory stimuli may be more likely to calm an agitated cat. When the investigation environment was a clinical setting, e.g., the examination room of a veterinary teaching hospital, cats were observed to be less stressed and easier to handle during physical examination when exposed to 10 min cat-specific music, compared to classical music or no music, in the absence of their carers [39]. Furthermore, when music was used as a means of reducing stress in hospitalized cats, both cat-specific and classical music had some beneficial effects, compared to no music (silence). More specifically, the cat-specific music group had a higher percentage of positive interactions with the researcher than the other treatments, while the classical music group exhibited a lower mean of RR in one of the assessments compared to the control treatment [40].

Overall, the results of studies in dogs are mixed, while the respective conclusions in cats seem to be more homogenous, although more limited. There are reports on dogs about the beneficial effects of music on stress and anxiety parameters, albeit not consistent between different conditions such as a rescue shelter or a veterinary clinic environment, or between different dog populations. Most studies reported a calming effect of classical music in potentially stressful environments such as boarding kennels, rescue shelters and veterinary clinics [29,30,31,33,48,49], while other studies reported no significant effect [37,38] or dogs’ preference for other music genres [33]. Furthermore, music specifically designed for dogs did not appear to have many beneficial effects over a random selection of classical music [30,32,34,37], while exposure to an audiobook seems to yield ambiguous results [32,48]. On the other hand, feline literature seems to be more explicit, albeit more limited, demonstrating that cat-specific and classical music can both promote calm behaviours and reduce stress and anxiety in different environments in cats [39,40,50].

## 5. Music’s Effect During the Perioperative Period

### 5.1. Literature Evidence on Humans

Apart from music’s beneficial effects on reducing parameters like stress and anxiety or pain in both healthy and diseased human populations, there have been reports supporting music’s effect, as an adjunctive treatment, on patient outcomes in different settings like intensive care units (ICU), or even during the perioperative period.

#### 5.1.1. ICU

Critically ill patients admitted to the ICU are exposed to a variety of stressful conditions, while an additional unpleasant and anxiety-producing situation seems to be the state of receiving mechanical ventilatory support. It has been reported that in approximately 70–80% of ICU patients, and especially in ventilator-dependent patients, significant levels of anxiety are observed, and sedatives and/or analgesics are routinely administered to alleviate distress and facilitate patient comfort [116]. Non-pharmacological approaches such as music therapy can mitigate stress response and decrease anxiety and pain during mechanical ventilation in critically ill patients, with a subsequent decrease in sedative requirements, leading to an accelerated ventilator weaning process and recovery [116]. A randomized controlled trial (RCT) including 373 patients on acute ventilatory support from 12 ICUs at five USA hospitals evaluated the effect of self-initiated patient-directed music on anxiety levels and sedation requirements. The critically ill subjects who were exposed to music during ventilatory support exhibited a 36.5% reduction in anxiety, and an approximately 40% reduction in sedation intensity and sedation frequency, compared to the standard care group [117]. Furthermore, a systematic review evaluating the effects of music on inflammatory biomarkers in intensive care and postoperative patients found that music listening may be associated with decreases in serum cortisol levels, demonstrating a potential decrease in the level of systemic stress and inflammation [118].

#### 5.1.2. Perioperative Period

Patients referred to surgery often experience significant perioperative anxiety, stress and fear [11,18] and, according to a systematic review and meta-analysis of 53 studies, anxiety was the psychological variable most frequently measured before surgery [20]. Another systematic review and meta-analysis including 14,652 patients (28 studies) concluded that approximately 50% of surgical patients experienced preoperative anxiety [21], and a more recent RCT also revealed similar rates; the overall incidence of preoperative anxiety in that RCT, which included women undergoing elective non-cardiac surgery, was 53.7% according to the validated scale that was used to determine anxiety [68]. Perioperative stress and preoperative anxiety can affect perioperative anaesthetic management and are associated with several negative clinical outcomes, including prolonged anaesthesia induction, increased anaesthetic requirements for induction and maintenance, haemodynamic instability, delayed recovery, postoperative delirium, potentiation of postoperative pain, increased postoperative analgesic consumption, immune system response impairment, higher risk of infection, wound healing delay, patient dissatisfaction, increased length of hospital stay and potentially increased health-care costs [11,20,21]. The systematic review of 53 studies, already mentioned above, found that perioperative anxiety, along with depression or pain catastrophizing, were classified as psychological correlates of acute postsurgical pain [20]. According to the US Institute of Medicine, approximately 80% of surgical patients in the US report postoperative pain, while 88% of these patients report moderate, severe or extreme pain levels [19]. Another prospective cohort study of 50,523 patients showed that even minor- to medium-level surgical procedures like appendectomy or tonsillectomy resulted in unexpectedly high levels of postoperative pain [119], which have been related to increased morbidity, functional and quality-of-life impairment, delayed recovery time, prolonged duration of opioid use and higher health-care costs [19].

To reduce patient anxiety and attenuate pain response during the perioperative period, a variety of drugs like sedatives, anxiolytics and opioid analgesics are typically administered. However, they often exhibit side effects and may interfere with smooth patient recovery or compromise the long-term quality of life [11]. Therefore, there is growing interest in non-pharmacological interventions, in terms of integrative medicine, as an adjunct to conventional procedures and pharmacotherapies for treating perioperative pain and anxiety, as part of a multimodal approach. More specifically, a consortium of multiple healthcare organizations in the USA, involved in surgical care, established seven guiding principles for acute perioperative pain management, and the third principle points out that clinicians should offer multimodal analgesia, or the use of a variety of analgesic medications and techniques combined with non-pharmacological interventions, for the treatment of postoperative pain in adults [24]. Music-based interventions, as an inexpensive, non-invasive, non-pharmacological adjunct, seem to be an effective approach for the perioperative control of pain, anxiety and reduction in sedative requirements in surgical patients, without reported side effects [7,11,12,14,17,18,27,28].

The first report of music as a non-pharmacological adjunct during the perioperative period was made in 1914; a phonograph was employed in the operating room as a means of patient-calming and distraction from the anxiety of the surgery during operations performed partially or entirely with local anaesthesia [120]. Thereafter, many RCTs and systematic reviews and meta-analyses have investigated the effect of music interventions during the perioperative period on different surgical populations and surgical procedures, evaluating several outcome measures. Most of the authors propose that sufficient research has been conducted to prove that perioperative music interventions should be available to all patients undergoing surgical operations, because of the observed beneficial impact on the evaluated outcome parameters [11,12,13,14,15,16,17,18,121].

Depending on the phase of the perioperative period that the musical intervention was implemented, a variety of outcomes (either stated as primary or secondary) affecting the prognosis of the patient have been measured. Music’s effect has been evaluated on outcome parameters like the patient’s neurohormonal stress response to surgery, the perioperative stress and anxiety levels, the sedation or general anaesthetic requirements, the pain levels, the analgesic requirements and other parameters related to patient prognosis.

There have been observed decreased plasma cortisol levels [69,74,122] and increased oxytocin levels [76] in patients exposed to music perioperatively, which are related to reduced stress response in patients. However, no effect on the neurohormonal stress response was observed in a study when music was applied to patients under general anaesthesia, according to epinephrine, norepinephrine, cortisol and adrenocorticotropic hormone (ACTH) blood level measurements [123].

##### RCTs Regarding the Effect of Music During the Perioperative Period

As already mentioned, patients referred to surgery often experience significant perioperative anxiety, stress and fear [11,18] and music interventions have been suggested to reduce the anxiety associated with the perioperative period [28,68,69,124,125,126]. Music had a beneficial effect on both preoperative anxiety [68,124,125], which was found comparable to benzodiazepines if not even greater [124,125], and postoperative anxiety after both minor and major surgery [28,69,126]. In the study of Wang et al. [68], the reduction in the preoperative anxiety levels after more than 30 min of preferred music listening resulted in attenuation of haemodynamic instability during anaesthesia induction, while the music-induced decrease in postoperative anxiety contributed to a significant reduction in systolic BP, HR and RR in patients after cardiac surgery [126].

The reduced anxiety levels that have been related to perioperative music interventions seem to correlate with increased sedation levels or a sparing effect on sedation requirements, as well. Patients who were exposed to their favourite music after midazolam administration displayed increased sedation levels and lower bispectral index (BIS) values compared to patients who were only treated with midazolam [127]. Furthermore, there have been reports of reduced sedative requirements both for midazolam [128] and propofol [74,129,130,131] in patients under regional anaesthesia. In the study of Koelsch et al. [74], propofol consumption to achieve light sedation was approximately 15% lower in the music group compared to the control group. However, no such general anaesthetic-sparing effect by music was demonstrated when the patients were under general anaesthesia [123,127,132,133].

The effect of music interventions on pain levels after surgery or on the postoperative opioid requirements has also been investigated when music was applied either during the intraoperative [92,134,135,136,137,138,139] or the postoperative period [6,91,126], or when music was employed on multiple moments perioperatively [28,69]. Music interventions were found to decrease both acute postoperative pain [126] and chronic pain, i.e., persistent postoperative pain [6], in patients after cardiac surgery. Another recent RCT demonstrated that patients undergoing minor surgery who received a music-based intervention perioperatively, as an add-on therapy to standard care, consumed 56.7% less opioids compared to patients not exposed to music therapy, with the highest effect being observed on the 1st postoperative day [28]. However, in other RCTs, no music effect was observed on postoperative pain or opioid consumption [123,138,139].

##### Systematic Reviews and Meta-Analyses Regarding the Effect of Music During the Perioperative Period

Apart from the plethora of RCTs that have been conducted to evaluate the effect of music interventions, some of which are mentioned above, a few systematic reviews and meta-analyses aimed to summarize these results and assess the quality of the body of literature in order to provide more robust clinical evidence about the effectiveness of music interventions during the perioperative period in humans. These systematic reviews evaluated the effects of music interventions on various outcomes like perioperative anxiety, neuroendocrine stress response to surgery, postoperative pain (pain scores), haemodynamic stability, intraoperative sedative requirements, postoperative (opioid) analgesic requirements, patient satisfaction or length of stay, in a wide variety of surgical procedures ranging from minor endoscopic interventions to more invasive surgical procedures such as abdominal, orthopaedic, cardiothoracic or transplantation surgery [11,12,13,14,15,16,17,18]. Bradt et al. [11] proposed that music interventions may provide a viable alternative to sedatives and anti-anxiety drugs for reducing preoperative anxiety, after evaluating 26 trials, including 2051 patients. The same music’s anxiety-reducing effect was also demonstrated perioperatively by subsequent systematic reviews including different surgical procedures [12,14] and after cardiothoracic surgery [18]. In the systematic review of Kuhlmann et al. [14] which included 92 RCTs (7385 patients), the lower anxiety levels in surgical patients were equivalent to a decrease of 21 mm on a 100 mm visual analogue scale (VAS). That beneficial effect of perioperative music on anxiety levels could be related to the reduced intraoperative propofol and midazolam requirements that were reported in the systematic review of Fu et al. [17]. Perioperative music listening has also been associated with attenuation of the neuroendocrine stress response to surgery which has been reflected in lower cortisol levels in patients postoperatively; however, the level of evidence was low either because of high methodological risk of bias or because of small sample sizes of the included studies [15,118]. Additionally, music has been proposed as a means of reducing postoperative pain or postoperative opioid consumption [12,14,16,17,18]. In a systematic review including 92 RCTs (7385 patients), music interventions before, during or after surgery significantly decreased pain in patients compared to controls, with a decrease equivalent to 10 mm on a 100 mm VAS [14]. Furthermore, another systematic review of 55 RCTs demonstrated significantly reduced postoperative opioid requirements both at 24 h and 3 days after surgery when either vocal or instrumental music was implemented perioperatively [17]. Overall, music therapy was found to have the greatest effect on pain when music was offered in the postoperative phase, and the greatest effect on anxiety when music was played during the preoperative phase; there were also significantly decreased pain levels when patients listened to music during regional anaesthesia [14]. Although preoperative anxiety or postoperative pain have been considered as factors that may determine morbidity, duration of hospital stay or even mortality, the systematic reviews that evaluated perioperative music’s effect on length of hospital stay did not demonstrate any beneficial effect, even though no adverse effects were reported [12,17].

##### Systematic Reviews and Meta-Analyses Regarding the Effect of Music Specifically During the Intraoperative Period, Under General Anaesthesia

Some of the aforementioned systematic reviews and meta-analyses found a beneficial effect of music even when applied solely intraoperatively, under general anaesthesia [12,14]. The sub-group analysis of perioperative music interventions under general anaesthesia revealed a statistically significant pain- and anxiety-reducing effect [12,14]. Music was found to significantly reduce pain levels, analgesic requirements and anxiety when provided intraoperatively under general anaesthesia; however, its effect was less profound compared to conscious patients [12]. Another systematic review, focusing exclusively on interventions performed under general anaesthesia, also found beneficial effects of music. This review, which included 610 patients undergoing elective surgeries, found that music therapy is a valuable intervention during general anaesthesia resulting in significant pain reduction, lower opioid consumption and increased patient satisfaction [13]. A more recent systematic review and meta-analysis evaluated the effect of auditory stimuli during general anaesthesia on postoperative patient outcomes and recovery, as well [121]. It included 53 RCTs (4200 patients); however, the intraoperative auditory interventions consisted not only of music but also of therapeutic suggestions, audiobooks or words. The authors observed a significant moderate to large beneficial effect of intraoperative music during general anaesthesia on postoperative pain and opioid requirements for the first 24 h after surgery, while therapeutic suggestions had no apparent effect on recovery. According to the authors, this systematic review and meta-analysis showed that intraoperative auditory stimuli can be perceived and processed during general anaesthesia, leading to implicit memory formation without explicit awareness. The current conclusions conform with the findings of previous studies which demonstrated that primary cortical responses to auditory stimulation are not totally suppressed, and patients may potentially process auditory information under deep sedation or even under general anaesthesia [140,141,142].

### 5.2. Literature Evidence on Dogs and Cats

Although there is plenty of literature on surgical human patients, studies in domestic animals concerning perioperative music implementation are scarce. To the authors’ knowledge, there are only six studies in dogs and cats that investigate the potential effect of music interventions during the perioperative period (Table 1) [41,42,43,44,45,46].

#### 5.2.1. Preoperative Period

Two of these studies investigated the potential benefit of music implementation during the preoperative period [43,44]. They were both conducted on dogs and evaluated music’s impact on preoperative parameters such as depth of sedation [43,44] or propofol requirements for the induction of anaesthesia [44]. Albright et al. [43] found that dogs were more sedated when exposed to lower-intensity auditory stimulation (40–60 dB) compared to high-intensity noise (80–85 dB) after 10 μg/kg intramuscular dexmedetomidine administration, meaning that the quality of sedation is negatively impacted by a loud and noisy environment even after administration of a potent sedative. Furthermore, they observed that exposure to music specifically designed for dogs (Through a Dog’s Ear) did not improve sedation; dogs exposed to that type of dog relaxation music at 45–50 dB were less sedated compared to when dogs were exposed to the background noise of 40–45 dB under dexmedetomidine premedication, according to spontaneous behaviour scores, concluding that the use of music specifically designed for dogs to improve dexmedetomidine sedative effects cannot be supported [43]. On the other hand, a more recent study found that dogs exposed to classical music (Chopin and Mozart) achieved higher sedation scores compared to the control silent condition after administration of a mild premedication protocol (acepromazine and butorphanol) [44]. According to the authors of the latter study, the reported music-induced level of sedation increase contributed to a 20% reduction in propofol dose requirements for tracheal intubation. However, differences regarding the methodology, and specifically on parameters such as the type of music (Through a Dog’s Ear vs. classical music), the features of the auditory stimulation (tempo, harmony, pitch, instrumentation), the volume of the auditory stimulation, the duration of the auditory stimulation, the type of the preoperative sedative regimen (dexmedetomidine vs. acepromazine) or the sample sizes, may have affected the results and contributed to the conflicting findings between the two studies.

#### 5.2.2. Intraoperative Period

Regarding the incorporation of music during the intraoperative period, veterinary literature includes three studies, thus far; two of them were conducted in cats [41,42] and one in dogs [46]. All the studies reported beneficial effects of intraoperative music interventions either under general anaesthesia in cats presented for elective ovariohysterectomy [41,42] or under a light anaesthetic plane (as monitored by BIS and neurological assessment) in dogs undergoing minor skin surgery [46]. Mira et al. [41,42] reported that exposure to music affects ANS activity by promoting parasympathetic activity predominance and indirectly concluded that cats are likely to preserve auditory sensory stimuli processing under anaesthesia. More specifically, they reported that cats under general anaesthesia not only seemed to prefer music compared to a silent scenario but appeared to prefer classical music compared to pop music and especially heavy metal music. These conclusions were based on reported physiological parameters alterations, which are subject to the control of the ANS, such as the RR and the pupillary diameter (PD) [41], or the HR and the systolic BP [42]. Most cats exhibited lower mean RR, PD, HR and systolic BP values when exposed to classical music, intermediate values to pop music and higher values to heavy metal music. A more recent prospective, blinded, crossover, randomized experimental study conducted in 20 laboratory dogs undergoing skin surgery under a light anaesthetic plane found no significant effect of classical music on physiological parameters (HR, RR or BP) intraoperatively [46]. However, the authors report that exposure to classical music exhibited an anaesthetic and analgesic sparing effect, by evaluating the intraoperative isoflurane and fentanyl requirements. Dogs were exposed to Chopin’s music, Mozart’s music or no music preoperatively (via loudspeakers) and intraoperatively (via headphones), while anaesthetic depth and intraoperative nociception level were monitored by BIS and neurological assessment, and by acute ANS responses, respectively. Dogs exposed to Chopin or Mozart music intraoperatively under a light anaesthetic plane (BIS ≈ 70) required significantly lower isoflurane and fentanyl concentrations compared to dogs in the no-music setting. The intraoperative fentanyl-sparing effect of music was further supported by the less pronounced alterations of substance P, a neuropeptide with a critical role in pain transmission, in dogs exposed to music compared to dogs in the no-music treatment. However, the results from the intraoperative studies in cats [41,42] and the results from the study of Georgiou et al. [46] in dogs cannot be directly comparable due to distinct differences in the study design. Although all three studies observed a beneficial effect of intraoperative music, the duration of the intraoperative music interventions, the targeted anaesthetic depth, the anaesthetic depth assessment methods and the sample sizes varied. More specifically, Mira et al. [41,42] applied 2 min music interventions while the respective music intervention duration in the study of Georgiou et al. [46] was approximately 90 min. Furthermore, the aforementioned studies in cats targeted a surgical anaesthetic depth, without mentioning the isoflurane concentrations used or the exact anaesthetic depth assessment method though, in contrary to the respective study in dogs where a light anaesthetic plane was used and the isoflurane concentrations and the method of the anaesthetic depth assessment were clearly stated. Finally, Mira et al. [41,42] used a sample of 12 female cats, while Georgiou et al. [46] used 20 dogs in a crossover design.

#### 5.2.3. Postoperative Period

One study in dogs investigated the effect of music specifically designed for dogs, as part of an integrative environmental enrichment approach, on the postoperative period [45]. Auditory stimulation with low-volume and low-tempo classical music (Through a Dog’s Ear) for at least 8 h per day was not the sole enrichment method applied, but multiple methods of environmental enrichment such as social enrichment (positive human interaction sessions), physical enrichment (dog-appeasing pheromones) and olfactory stimulation (lavender and chamomile oil) were employed concurrently, complementary to the standard postoperative protocol, in dogs undergoing hemilaminectomy due to acute intervertebral disc extrusion. This randomized, double-blinded, placebo-controlled study aimed to evaluate the effect of different methods of environmental enrichment primarily on postoperative pain (postoperative opioid requirements) and secondarily on postoperative anxiety and return of appetite, compared to the standard postoperative environment. It was found that dogs recovering in an environmentally enriched quiet room received fewer doses of rescue analgesia during the first 24 h postoperatively and consumed more food in the 48 h postoperative period compared to dogs recovering in the standard environment of an intensive care room. Although no difference was observed regarding the requirements of trazodone, as an anti-anxiety medication, the higher number of meals consumed postoperatively was associated with lower anxiety levels, as anorexia can be considered a sign of anxiety in a hospital setting. However, the small sample size, the fact that different surgeons performed the surgical procedures, the subjectivity of the pain and anxiety assessment and the fact that multiple methods of environmental enrichment were evaluated at the same time during the postoperative period were recognized by the authors as limitations of the study.

## 6. Clinical Implications and Future Directions

While music is widely regarded as a non-invasive and low-risk intervention, especially compared to pharmacological approaches, it may not be entirely without potential limitations. Unsuitable genres, volumes, tempos or delivery methods may cause overstimulation or aversion in some animals. Furthermore, in clinical environments music may interfere with communication or concentration among the staff or prove unrealistic in already noisy settings. Therefore, careful selection and application of music are essential to optimize its benefits and minimize undesired phenomena. Furthermore, there has been observed in some cases that behavioural indicators of reduced stress do not align with physiological measures. This tension underscores the multifaceted nature of stress and the limitations of relying on single-dimensional outcome measures.

Another fact to consider is the species-specific differences in auditory perception between humans, dogs and cats, which could influence the effectiveness of music as a perioperative intervention. Although species differences in auditory range are documented (humans perceive frequencies between 20 Hz and 20 kHz, while dogs and cats exhibit a wider hearing range of approximately 65 Hz to 45 kHz and 48 Hz to 85 kHz, respectively) [111,143], comparative analyses of auditory perception (regarding music-specific hearing thresholds or tempo processing) in dogs and cats are not extensively represented in veterinary literature. So, the extent to which human evidence can be directly applied to veterinary patients should be carefully considered and these differences highlight the importance of tailoring auditory stimuli to each species to optimize perioperative outcomes.

Although veterinary evidence remains preliminary, existing studies suggest that music may serve as a low-cost, non-invasive, non-pharmacological tool to support perioperative care as part of a multimodal approach, particularly in reducing patients’ perioperative anxiety and stress or enhancing their recovery. Music could be introduced in the perioperative period of dogs and cats to potentially provide additional anxiolysis, analgesia or optimize the final surgical outcome. Species-specific preferences concerning genre, tempo, pitch, instrumentation and volume of exposure should be considered, while music should not be interfered with perioperative clinical monitoring or staff communication. Additionally, music interventions should be individualized, recognizing that not all veterinary patients may benefit equally, taking into account the potential individual aversion to music or the species-specific differences in auditory perception between dogs and cats.

Future studies should focus on standardized RCTs assessing physiological, behavioural or even neurobiological parameters, using well-described music protocols (e.g., genre, tempo, pitch, instrumentation, volume, method of delivery, duration and timing of intervention) and consistent outcome measures to provide a more comprehensive image of therapeutic outcomes. Investigating the components of a music intervention will be critical to potentially incorporate them into the perioperative veterinary practice successfully or even distinguish interspecies differences. Finally, feasibility, staff feedback and patient safety should be assessed and supported.

## 7. Conclusions

The current review aimed to provide the physiological background of music interventions and an insight into the potential role of music during the perioperative period of dogs and cats, according to the existing literature both in humans and domestic animals. The studies regarding perioperative music implementation in dogs and cats are limited; however, their results seem quite promising. Music as a safe, low-cost, low-risk, non-invasive, non-pharmacological adjunctive strategy could greatly expand treatment options for veterinary patients during the perioperative period, in terms of a multimodal approach, improving their overall experience and potentially enhancing the final surgical outcome and welfare. However, music is a highly variable and complex intervention, and the existing research methods are characterized by heterogeneity that could compromise replicability. Differences in animal species, surgical procedures, type of musical stimuli (genre, tempo, volume), delivery methods, timing and duration of the intervention, sample sizes and outcome measures contribute to this heterogeneity and hinder direct comparison and synthesis. Despite promising early evidence, this research area remains underexplored, and this gap highlights the need for targeted, species-specific investigations in clinical contexts. So, it goes without saying that a feasible framework for an effective music intervention in dogs and cats needs to be defined in view of its potential application in the veterinary clinical practice. Considering that music seems to exert physiological effects and affect animals similarly to humans, more adequately powered RCTs including clearly stated research questions and well-defined means of outcome measure evaluation need to be conducted, to standardize music protocols.

## Figures and Tables

**Table 1:** Summary of studies evaluating the effect of music during the perioperative period in dogs and cats.

Study (Author, Year)	Species	Surgical Intervention	Type of Music (Genre)	Tempo (BPM ^1^)	Volume (dB ^2^)	Delivery Method	Timing of Intervention	Duration of Intervention	Sample Size	Outcome Measures	Key Findings
Mira et al., 2016a [41]	cat	Ovariohysterectomy	1. Classical 2. Pop 3. Heavy metal	Not reported	<80 dB	Head-phones covering the whole ear	Intraoperatively (under general anaesthesia)	8 min in total (2 min of each condition)	12 female cats	1. Respiratory rate (RR ^3^) 2. Pupillary diameter (PD ^4^)	1. Cats under general anaesthesia perform auditory sensory stimuli processing 2. Music-dependent RR, PD responses, associated with ANS ^5^ activity
Mira et al., 2016b [42]	cat	Ovariohysterectomy	1. Classical 2. Pop 3. Heavy metal	Not reported	<80 dB	Head-phones covering the whole ear	Intraoperatively (under general anaesthesia)	8 min in total (2 min of each condition)	12 female cats	1. Heart rate (HR ^6^) 2. Systolic blood pressure (SBP ^7^)	1. Cats under general anaesthesia perform auditory sensory stimuli processing 2. Music-dependent HR, SBP responses, associated with ANS activity
Albright et al., 2017 [43]	dog	No surgery	Specifically designed for dogs (Through a Dog’s Ear)	Low-tempo music (approx. 50–60 BPM)	40–85 dB	Speakers	Preoperatively	20 min	10 dogs	Depth of sedation (spontaneous behaviour scoring, accelerometry, restraint test scoring)	1. Noise up to 85 dB has negative impact on sedation 2. Music did not improve dexmedetomidine sedative effects
Georgiou et al., 2023 [44]	dog	No surgery	Classical (Chopin, Mozart)	Slow-tempo excerpts (lento sostenuto, andante)	50–65 dB	Speakers	Preoperatively	90 min	20 dogs	1. Depth of sedation 2. Induction propofol requirements	1. Music contributed to increased depth of sedation 2. Music contributed to 20% lower propofol requirements
Pennington et al., 2023 [45]	dog	After hemilaminectomy surgery	Specifically designed for dogs (Through a Dog’s Ear)	Low-tempo music (approx. 50–60 BPM)	Not mentioned	Not mentioned	Postoperatively	8 h per day for 48 h	20 dogs	1. Postoperative pain (pain scores, rescue opioid requirements) 2. Level of postoperative anxiety (trazodone requirements, number of meals consumed)	1. Lower postoperative rescue methadone requirements for the first 24 h postoperatively in dogs recovering in an enriched environment 2. The same dogs consumed more food in the first 48 h postoperatively, with that being attributed to lower anxiety levels
Georgiou et al., 2024 [46]	dog	Skin surgery	Classical (Chopin, Mozart)	Slow-tempo excerpts (lento sostenuto, andante)	65 dB	Headphones covering the whole ear	Intraoperatively (under light-plane anaesthesia)	Approximately 90 min	20 dogs	1. Intraoperative anaesthetic requirements (isoflurane concentrations) 2. Intraoperative analgesic requirements (fentanyl requirements) 3. Substance-P concentrations 4. Physiologic variables	1. Isoflurane- and fentanyl-sparing effect of intraoperative music in dogs under light-plane anaesthesia 2. Rapid increases in Substance-P concentrations after skin incision, being related to acute pain

^1^ BPM: beats per minute ^2^ dB: decibel ^3^ RR: respiratory rate ^4^ PD: pupil dilation ^5^ ANS: autonomic nervous system ^6^ HR: heart rate ^7^ SBP: systolic blood pressure.

## Data Availability

The data presented in this study are available on request from the corresponding author.

## Abbreviations

The following abbreviations are used in this manuscript:

AC	auditory cortex
ACC	anterior cingulate cortex
ANS	autonomic nervous system
BIS	bispectral index
BP	blood pressure
CHF	congestive heart failure
dB	decibel
EEG	electroencephalography
fMRI	functional magnetic resonance imaging
HPA	hypothalamic–pituitary–adrenal
HR	heart rate
HRV	heart rate variability
ICU	intensive care unit
kHz	kilohertz
MEG	magnetoencephalography
PAG	periaqueductal gray
PD	pupillary diameter
PET	positron emission tomography
RCT	randomized controlled trial
RR	respiratory rate
SNS	sympathetic nervous system
VAS	visual analogue scale
